# Inhibition of the AKT pathway in cholangiocarcinoma by MK2206 reduces cellular viability via induction of apoptosis

**DOI:** 10.1186/s12935-015-0161-9

**Published:** 2015-02-04

**Authors:** Jacob M Wilson, Selvi Kunnimalaiyaan, Muthusamy Kunnimalaiyaan, T Clark Gamblin

**Affiliations:** Department of Surgery, Division of Surgical Oncology, Medical College of Wisconsin, 9200 W Wisconsin Ave, Milwaukee, WI 53226 USA; Medical College of Wisconsin, Translational and Biomedical Research Center, 8701 Watertown Plank Road, Milwaukee, WI 53226 USA

**Keywords:** Cholangiocarcinoma, MK2206, PI3K/mTOR/AKT, Apoptosis

## Abstract

**Introduction:**

Cholangiocarcinoma (CCA) is an aggressive disease with limited effective treatment options. The PI3K/Akt/mTOR pathway represents an attractive therapeutic target due to its frequent dysregulation in CCA. MK2206, an allosteric Akt inhibitor, has been shown to reduce cellular proliferation in other cancers. We hypothesized that MK2206 mediated inhibition of Akt would impact CCA cellular viability.

**Study methods:**

Post treatment with MK2206 (0-2 μM), cellular viability was assessed in two human CCA cell lines—CCLP-1 and SG231—using an MTT assay. Lysates from the MK2206 treated CCA cells were then examined for apoptotic marker expression levels using Western blot analysis. Additionally, the effect on cellular proliferation of MK2206 treatment on survivin depleted cells was determined.

**Results:**

CCLP-1 and SG231 viability was significantly reduced at MK2206 concentrations of 0.5, 1, and 2 μM by approximately 44%, 53%, and 64% (CCLP-1; p = 0.01) and 32%, 32%, and 42% (SG231; p < 0.00005) respectively. Western analysis revealed a decrease in AKT^Ser473^, while AKT^Thr308^ expression was unchanged. In addition, cleaved PARP as well as survivin expression increased while pro-caspase 3 and 9 levels decreased with treatment. Depletion of survivin in CCLP-1 cells resulted in apoptosis as evidenced by increased cleaved PARP. In addition, survivin siRNA further enhanced the antitumor activity of MK2206.

**Conclusions:**

This study demonstrates that by blocking phosphorylation of Akt at serine473, CCA cellular growth is reduced. The growth suppression appears to be mediated via apoptosis. Importantly, combination of survivin siRNA transfection and MK2206 treatment significantly decreased cell viability.

## Introduction

Cholangiocarcinoma (CCA) is a rare but aggressive malignancy arising from the epithelium of either the intrahepatic (ICC) or extrahepatic (ECC) biliary ducts [1,2]. While cholangiocarcinoma represents only 3% of all gastrointestinal malignancies, it is the second most common primary hepatic lesion trailing only hepatocellular carcinoma (HCC) [1-3]. Despite its rarity, incidence of intrahepatic CCA has been rising internationally and it has overtaken HCC as the most lethal primary liver cancer in England and Whales [4,5].

Currently, surgical resection offers the only curative treatment for CCA [1,3,4]. However, the majority of patients present with unresectable disease and even in those achieving a R0 resection, 5-year survival rates remain low (22-35% [3,4,6-8]) with greater than 50% recurrence at a median 31-month follow-up [8]. In unresectable cases, transarterial chemoembolization (TACE) (gemcitabine with cisplatin or oxaliplatin) has been shown to improve survival but only to a median survival of approximately 14 months [9]. Liver transplantation in unresectable cases is another option in highly selected patients, but is limited due to scarcity of donors [3,10]. Overall, the median survival without treatment is reported to be only 5–8 months, with 5-year survival at <5% [1,9,11]. Therefore, because the prognosis for CCA is dismal and no systemic therapy has demonstrated substantial efficacy [9], there exists an urgent need for identification of novel therapeutic targets.

The phosphatidylinositol 3-kinase (PI3K)/protein kinase B (Akt)/mTOR pathway is an attractive therapeutic target as it is involved in cellular survival, proliferation, metastasis, metabolism, and angiogenesis [11-14]. The dysregulation of this pathway has been implicated in the pathogenesis of many cancer types, including CCA [11-15]. Studies have demonstrated that increased expression of phosphorylated Akt is present in >80% of extrahepatic CCA [12] and >60% of intrahepatic CCA [13,16]. Therefore, inhibition of this pathway could prove therapeutic.

MK2206, a novel, small molecule, allosteric Akt inhibitor has demonstrated promising efficacy in vitro and in early clinical trial [11,15,17,18]. While several Akt inhibitors have been developed, many have been plagued by either non-specificity, unacceptable toxicities, or have lacked anti-tumor efficacy *in vivo* [19]. Thus far, MK2206 has demonstrated moderate single agent anti-tumor efficacy with acceptable toxicity in humans [17]. Despite one previous report of MK2206 use in cholangiocarcinoma, the mechanism of action in these cells needs significant clarification [11].

This study sought to examine the effects of MK2206 on two human CCA cell lines—CCLP-1 and SG231. The effects of MK2206 on CCA cellular proliferation were examined and the mechanism of cellular growth inhibition was identified by Western blot analysis. By inhibiting Akt phosphorylation via MK2206, it was hypothesized that CCA cellular proliferation would be reduced via induction of apoptosis.

## Materials and methods

### Cell culture and treatment

CCLP-1 and SG231 are human, intrahepatic cholangiocarcinoma cell lines (courtesy of Dr. Anthony J. Demetris, University of Pittsburgh, Pittsburgh, PA). CCLP-1 cells were maintained in Dulbecco Modified Eagle Medium (Sigma-Aldrich, St. Louis, Missouri, USA) supplemented with 10 mM HEPES (Life Technologies, Grand Island, New York, USA), 100 IU/ml penicillin, 100 μg/ml streptomycin (Life Technologies), 1× non-essential amino acids (Life Technologies), and 10% Fetal Bovine Serum (Life Technologies). SG231 cells were maintained in Minimal Essential Medium Alpha Medium (Life Technologies) supplemented with 10% Fetal Bovine Serum (Life Technologies). Both cell lines were grown in a humidified incubator at 37°C and 5% CO_2_. MK2206 was dissolved in dimethyl sulfoxide (DMSO; Sigma-Aldrich). Cells were then treated with varying concentrations of MK2206.

### Cellular viability

CCLP-1 and SG231 cellular viability was determined using a 3-(4,5-dimethylthiazol-2-yl)-2,5-diphenyltetrazolium bromide (MTT; Sigma-Aldrich) colorimetric assay. CCLP-1 and SG231 cells were plated in 24-well plates and left overnight to adhere. Cells were then treated at indicated concentrations of MK2206 in quadruplicates. Cell media with corresponding MK2206 concentrations was replenished at the 48 hour time interval. At 96 hours, media was replaced with 250 μL of RPMI media (Life Technologies) containing 0.5 mg/ml MTT. The cells were then incubated for 3.5 hours at which point 750 μl DMSO was added to each well. After another 5-minute incubation, absorbance was measured at 540 nm using a spectrophotometer (TECAN Infinite M200 PRO, San Jose, CA).

#### Small Interfering RNA transfection

For the siRNA transfection, CCLP-1 cells were plated into either 60 mm or 100 mm plates and allowed to grow over night. Then the cells were transiently transfected with non-specific, no target, control siRNA or survivin siRNA for 48 hours using Lipofectamine (Invitrogen, Carlsbad, CA, USA). The cells were subsequently prepared for use in further experiments. For the cell proliferation assay, the siRNA transfected cells from 100 mm plates were collected, counted and plated on a 48 well plate in quadruplicates. The following day, the cells were treated with DMSO (control) or MK2206 (0.5 and 1 μM) for an additional 48 hours. Cellular viability was measured as described above.

### Western blot analysis

After treatment with varying concentrations of MK2206 for 96 hours, CCA cells were lysed in RIPA buffer(Thermo Fisher) [15,20]. The total cellular protein concentrations were then quantified using a bicinchoninic acid (BCA) assay (Pierce, Rockford, Illinois, USA). After quantification, 30 μg of denatured protein was loaded onto 7.5, 10, 12, or 4-15% SDS-PAGE gels (Bio-Rad Laboratories, Hercules, CA, USA). The protein was then transferred to a nitrocellulose membrane (Bio-Rad) using a Trans-Blot Turbo (Bio-Rad). The membranes were subsequently blocked in PBS-T containing milk (1× PBS, 5% dry milk, 0.05% Tween-20) for 30–60 minutes. The blocked membranes were then incubated overnight at 4°C in their respective primary antibodies. The antibodies were diluted as follows: phosphorylated-AktSer473, total-PARP, cleaved PARP, (1:1000; Cell Signaling Technology, Beverly, Massachusetts, USA), cyclin D1, survivin and total Akt (Santa Cruz Biotechnology, Santa Cruz, CA, USA) were diluted 1:1000, 1:500, and 1:2000 respectively. Caspase-3, Caspase-9, and phosphorylated-AktThr308 (Cell Signaling Technology) were diluted 1:1000. In addition, glyceraldehyde-3-phosphate dehydrogenase (GAPDH; Santa Cruz Biotechnology) was diluted 1:3000. After overnight incubation in primary antibody, membranes were then washed 3 times for 5 minutes with PBS-T wash buffer (1 × PBS, 0.05% Tween 20). Membranes where then incubated for one hour in either anti-rabbit or anti-mouse secondary antibody (Santa Cruz Biotechnology; 1: 4000 dilution) depending on the source of the primary antibody. Following incubation, membranes were washed 3 times for 5 minutes each time in PBS-T wash buffer and then developed using SuperSignal West Dura, Femto (Thermo Scientific, Waltham, MA, USA), Immunstar, or Clarity (Bio-Rad) kits. Molecular Images ChemiDoc XRS^+^ imager with image lab software (Bio-Rad) was used to visualize the intensity of bands according to manufacturer’s instructions. Following development, band intensities were quantified using ImageJ software (NIH, Bethesda, USA) as needed.

### Statistical analysis

Unless otherwise noted, all results represent mean ± SEM. Significance between treatments was determined using SPSS statistics software (International Business Machines Coporation (IBM), Armonk, New York, USA; Oneway ANOVA). P-values of less than 0.05 were considered significant.

## Results

### MK2206 treatment reduces Cholangiocarcinoma cellular proliferation in a dose and time dependent manner

**Figure 1:**
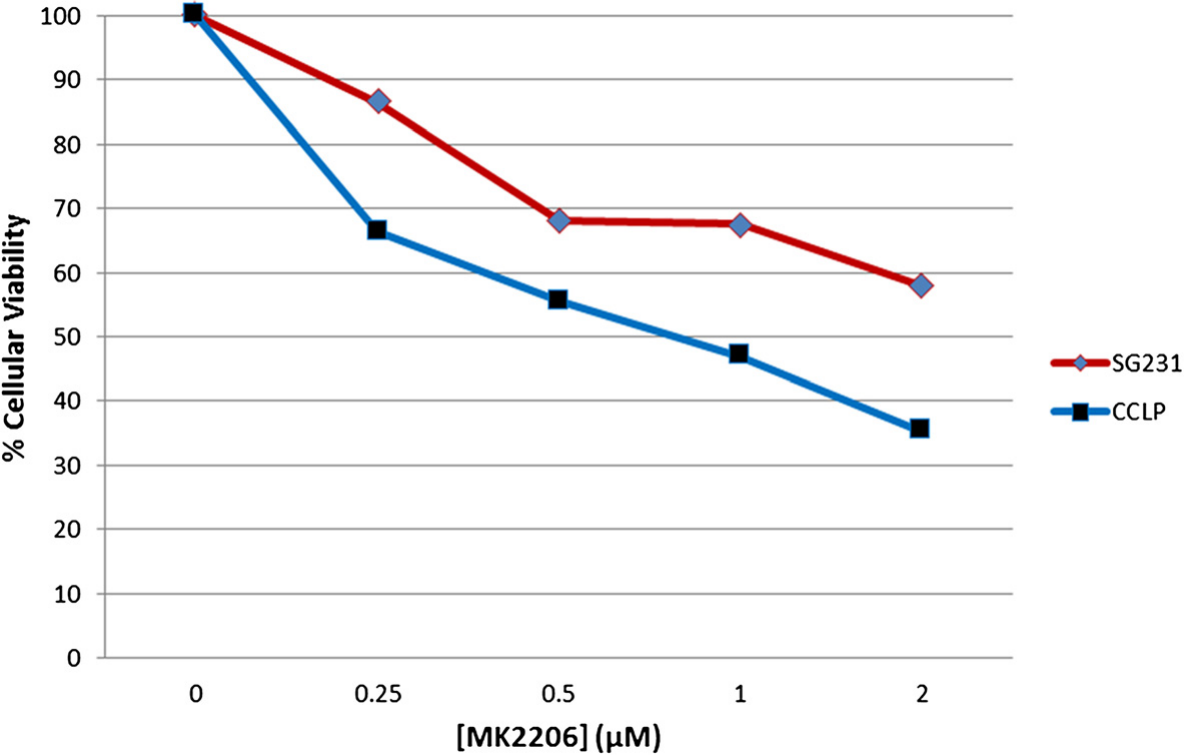
**Dose-dependent growth reduction upon MK2206 treatment.** MTT assay was used to determine cellular viability after 96 hours of treatment with MK2206 at the indicated doses. Growth reduction was significant in both cell lines at 0.5, 1, and 2 μM (p < 0.05).

**Figure 2:**
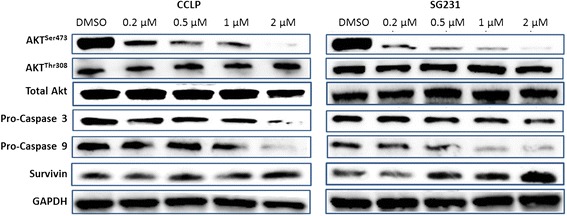
**MK2206 specifically inhibits phosphorylation at Serine 473 in CCA cells, leading to induction of apoptosis.** After 96 hours of MK2206 treatment at the indicated doses, CCLP-1 and SG231 cells were lysed and Western blot analysis was used to determine expression levels of phosphorylated AKT and total AKT in addition to apoptotic markers (Pro-Caspase 3, Pro-Caspase 9, and Survivin).

Cell viability results, as measured by MTT assay, indicated a dose and time correlate for growth reduction in both the CCLP-1 and SG231cell lines (Figure 1). After 48 hours, CCLP-1 and SG231 viability was reduced on average by 39% and 27% respectively (DMSO v. 2 μM; p < 0.0005 and p = 0.001 respectively). At 96 hours, CCLP-1 viability was significantly reduced at MK2206 concentrations of 0.5, 1, and 2 μM by approximately 44%, 53%, and 64% respectively (p = 0.01). SG231 also demonstrated significant reduction in viability of 32%, 32%, and 42% respectively for the same treatment concentrations (p < 0.0005) (Figure 2).

### MK2206 reduces levels of phosphorylated Akt^Ser473^ in CCLP-1 and SG231 cells

**Figure 3:**
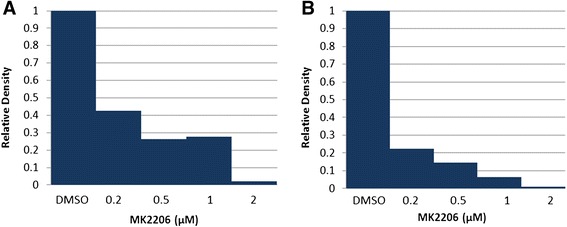
**Quantification of Akt** ^**Ser473**^ **western blot bands using ImageJ software. A)** CCLP **B)** SG231. Inhibition of Akt phosphorylation at Serine 473 by MK2206 is approximately dose dependent.

In order to confirm MK2206’s mechanism of action and the previously reported specificity [21], levels of Akt^Ser473^, Akt^Thr308^, and total Akt expression were examined by Western blot analysis. In both CCLP-1 and SG231 cell lines, Akt^Ser473^ was significantly reduced in MK2206 treated cells (0.1-2 μM; Figure 2). Quantification of the intensity of bands revealed a dose dependent reduction in expression of Akt^Ser473^ in both cell lines with a plateau between 0.5-1 μM MK2206 treatment levels in CCLP-1 (Figure 3A, B). Ultimately, treatment resulted in a reduction in levels of Akt^Ser473^ by >95% in both cell lines (DMSO v. 2 μM MK2206) whereas expression levels of Akt^Thr308^, the other phosphorylation site for Akt activation, were not changed by MK2206 treatment. Additionally, treatment did not affect the level of total Akt expression in cells, confirming the mechanism of MK2206 action to be specific inhibition of Akt^Ser473^ phosphorylation (Figure 2).

### Apoptosis induction accounts for the reduction in cellular proliferation upon MK2206 treatment

**Figure 4:**
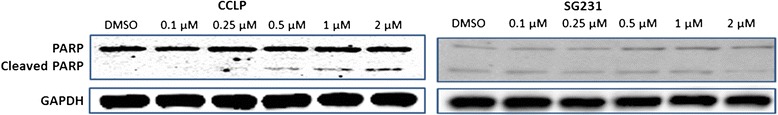
**MK2206 treatment induced cleavage of PARP in the CCLP cell line, confirming the mechanism of growth inhibition appears to be via apoptosis induction.** At the low concentrations used in this study, PARP and Cleaved PARP expression levels were not affected by MK2206 treatment in the SG231 cell line.

Western blot analysis was used to examine expression levels of full-length PARP, cleaved PARP, Caspase 3 and 9, and survivin in order to determine the effect of Akt inhibition on CCA. In CCLP-1, while full-length PARP expression was not noticeably different upon Akt inhibition, cleaved PARP increased in a dose dependent manner with expression first noted at the 0.25 μM MK2206 treatment level. However, in SG231, both full-length and cleaved PARP were equally expressed at all treatment levels (Figure 4). In both CCLP-1 and SG231, pro-caspase 3 and 9 were reduced significantly, indicating that drug treatment is leading to the cleavage of these proteins and inducing an apoptotic process (Figure 2). Interestingly, survivin, an anti-apoptotic protein was unexpectedly up-regulated in a dose dependent manner upon MK2206 treatment (Figure 2).

### Effect of survivin siRNA transfection on cell viability in CCLP-1

**Figure 5:**
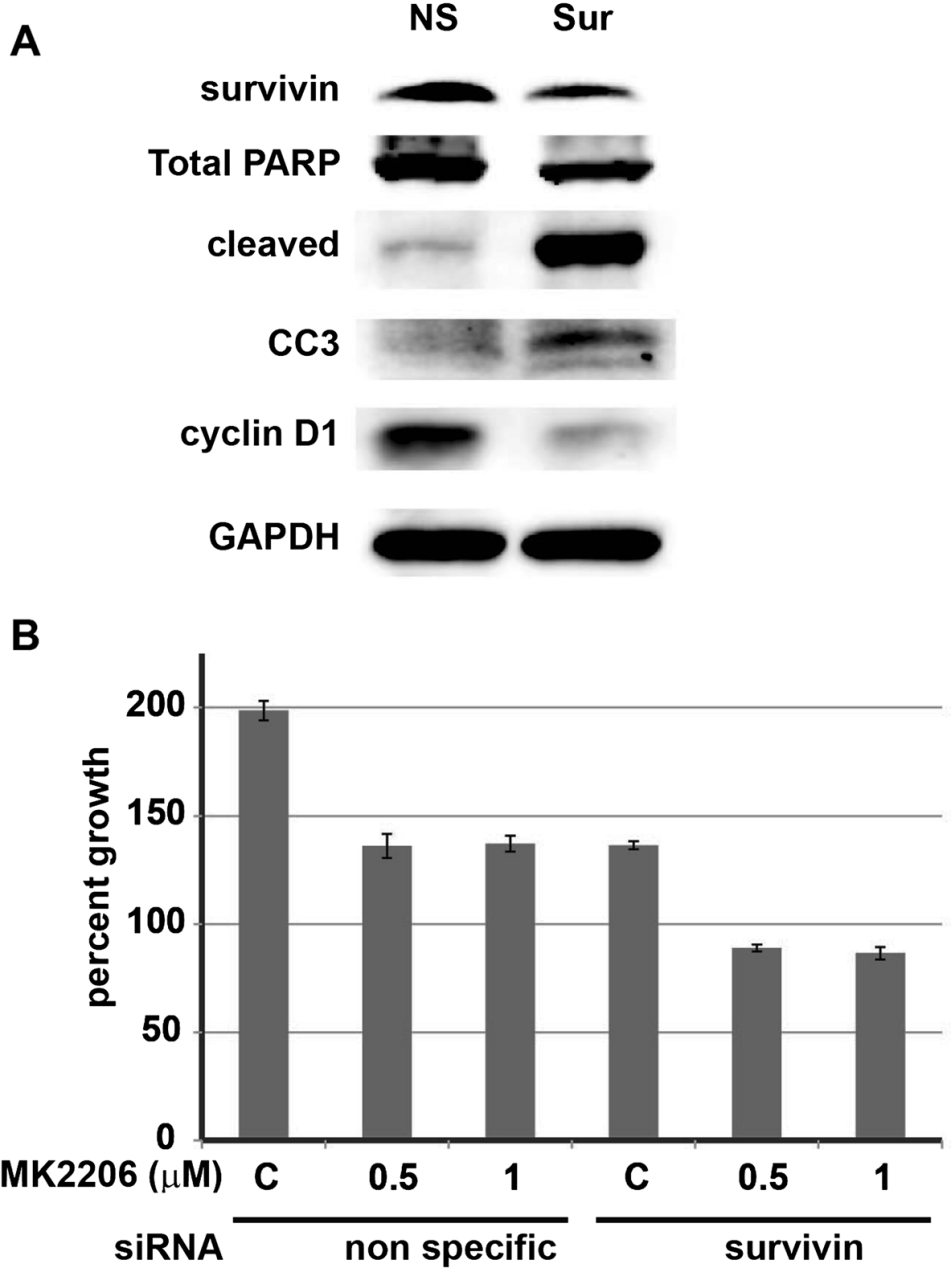
**Effects of survivin siRNA transfection and combined MK2206 treatment on CCLP-1 cells. A)** Survivin siRNA transfection resulted in apoptosis. **B)** Combined treatment of MK2206 in survivin siRNA transfected cells showed significant reduction in cell viability compared to non-specific (NS) siRNA plus MK2206 treatment.

Survivin siRNA was used to determine the effect on apoptosis in CCLP-1 cells. Survivin siRNA transfection resulted in induction of apoptosis as evidenced by the increased expression of cleaved PARP, cleaved caspase-3 whereas decreased expression of cyclin D1 and total PARP proteins compared to nonspecific siRNA transfected cells (Figure 5A). Importantly, survivin siRNA decreased the viability of CCLP-1 cells compared to control siRNA transfected group (Figure 5B). However, survivin siRNA transfection combined with MK2206 treatment of 0.5 and 1 μM for 48 hours significantly decreased cell viability (p = 0.01, compared with MK2206 alone; Figure 5B).

## Discussion

Cholangiocarcinoma represents a devastating malignancy that is plagued clinically by late detection and poor overall prognosis. Patients are often not surgical candidates but even with margin negative resection, most patients will experience recurrence and the prognosis is dismal [3,4,6-8]. Therefore, the majority of patients require systemic therapy, but to date no available therapy has proved especially efficacious [9]. The lack of available therapies necessitates the identification of novel therapeutic targets. The present study investigates the role of one such target: the PI3K/Akt/mTOR pathway.

The PI3K/Akt/mTOR pathway plays an important role in CCA pathogenesis as evidenced by its reported over activation in both intra and extrahepatic cholangiocarcinoma [12,13,16]. This, in addition to the known functions of Akt and a previous report demonstrating apoptosis induction upon Akt knockdown, indicates that Akt inhibition could be a potential therapeutic target [11-14,21]. Despite evidence that inhibition of all three Akt isoforms is necessary for maximal apoptosis induction, it is thought that isoform specific inhibition may mitigate toxicity in vivo [19,21]. Previous reports have shown that MK2206 may have Akt isoform specificity for Akt 1 and 2, which is consistent with the relative tolerability of MK2206 in clinical trial [17,18].

Previous studies [11,15,18,22,23], have demonstrated the efficacy of MK2206 as a single agent in other cancer types or in synergistic combination with RAD001 (an mTOR inhibitor) in cholangiocarcinoma cells in vitro. Ewald et al. [11] demonstrated an increase in p27, a cell cycle inhibitor, upon MK2206 treatment in two of three CCA cell lines (EGI-1, TFK-1, and SK-ChA-1 cell lines). While some induction of cell cycle arrest in CCA cells upon MK2206 treatment was observed, apoptotic markers were not fully investigated [11].

Examination of protein expression levels post-MK2206 treatment in the present study revealed that MK2206 specifically inhibits phosphorylation at AKT^Ser473^. In CCLP-1 and SG231 cholangiocarcinoma cell lines, AKT^Thr308^ and total AKT expression levels were not affected by drug treatment, confirming drug specificity. AKT^Ser473^ phosphorylation inhibition occurred in a dose dependent manner resulting in a dose dependent reduction in cellular viability at 48 and 96 hours in both cell lines. Importantly, the concentrations needed for growth reduction in CCA are less than those needed in other cancer types. It is anticipated that these concentrations could be achieved in vivo with limited toxicity.

The results of this study indicate that MK2206 is effective in reducing CCA cellular proliferation via the induction of apoptosis. Interestingly, survivin was up regulated in both CCLP-1 and SG231 upon MK2206 treatment. This is an intriguing finding given survivin’s established anti-apoptotic function. It is speculated that compensatory activation of survivin could be mitigating MK2206 mediated apoptosis induction. In addition, a recent report on MK2206 use in hepatocellular carcinoma identified survivin as an important downstream mediator upon MK2206 treatment, and found increased apoptosis and reduced viability when survivin knockdown was added to the treatment regimen [24]. Given these findings, we used siRNA knockdown of survivin in order to determine the role of survivin in MK2206 treatment. The combination of survivin siRNA transfection and MK2206 treatment resulted in significant reduction in cell viability when compared to MK2206 treatment alone. These results suggest that the inhibition of survivin may sensitize the CCLP-1 cells to MK2206 treatment by preventing the activation of pro-survival proteins.

In the present study, we have demonstrated the effectiveness of MK2206 in inducing apoptosis as a monotherapy in CCA cell lines in vitro. Future work will investigate MK2206 use in vivo and will center on developing more effective combination therapies. Synergistic combination treatments are particularly promising as Akt pathway activation has been shown to mitigate chemotherapy and radiotherapy efficacy in treating CCA [25-29]. The known chemo-resistance of CCA may be attributable, at least in part, to Akt pathway activation [25]. Previous work has demonstrated enhanced sensitivity to treatment with oxaliplatin, gemcitabine and radiotherapy upon PI3K/mTOR/AKT inhibition and conversely, has demonstrated increased resistance to treatment with Akt activation [25-29]. Lastly, autophagy may be playing an important role, as PI3K/mTOR/Akt pathway inhibition has been shown to increase autophagy in CCA cells. Similarly, the nutritionally stressed and hypoxic microenvironment of tumors in vivo could also lead to increased autophagy. Importantly, inhibition of autophagy in CCA has been shown to induce apoptosis and increase chemosensitivity [30,31]. Future work should investigate the efficacy of combining MK2206 with other chemotherapy agents as well as clarifying the role of autophagy in this process.
